# Putative resistance gene markers associated with quantitative trait loci for fire blight resistance in *Malus* ‘Robusta 5’ accessions

**DOI:** 10.1186/1471-2156-13-25

**Published:** 2012-04-03

**Authors:** Susan E Gardiner, John L Norelli, Nihal de Silva, Gennaro Fazio, Andreas Peil, Mickael Malnoy, Mary Horner, Deepa Bowatte, Charmaine Carlisle, Claudia Wiedow, Yizhen Wan, Carole L Bassett, Angela M Baldo, Jean-Marc Celton, Klaus Richter, Herb S Aldwinckle, Vincent GM Bus

**Affiliations:** 1 The New Zealand Institute for Plant & Food Research Limited (PFR) Palmerston North, Private Bag 11600, Manawatu Mail Centre, 4442, Palmerston North, New Zealand; 2 USDA-ARS, Appalachian Fruit Research Station, 2217 Wiltshire Rd., Kearneysville, WV, 25430, USA; 3 PFR Mt Albert, Private Bag 92169, Auckland Mail Centre, 1142, Auckland, New Zealand; 4 USDA-ARS, Plant Genetic Resources Unit, 630W. North St., Geneva, NY, 14456, USA; 5 Julius Kühn-Institut (JKI), Institute for Breeding Research on Horticultural and Fruit Crops, Pillnitzer Platz 3a, D-01326, Dresden, Germany; 6 Foundation E. Mach - Istituto Agrario San Michele all'Adige, Via E. Mach 1, 38010, San Michele all'Adige, TN, Italy; 7 PFR Hawke’s Bay, Private Bag 1401, 4157, Havelock North, New Zealand; 8 Apple Research Center, College of Horticulture, Northwest A&F University, Yangling, Shaanxi, 712100, China; 9 UMR Génétique et Horticulture (GenHort), INRA ⁄ Agrocampus-ouest ⁄ Université d’Angers, Centre Angers-Nantes, 42 rue Georges Morel – BP 60057, 49071, Beaucouze´ Cedex, France; 10 JKI, Institute for Resistance Research and Stress Tolerance, Erwin-Baur-Str. 27, D-06484, Quedlinburg, Germany; 11 Department of Plant Pathology and Plant-Microbe Biology, Cornell University, 630W. North St., Geneva, NY, 14456, USA

## Abstract

**Background:**

Breeding of fire blight resistant scions and rootstocks is a goal of several international apple breeding programs, as options are limited for management of this destructive disease caused by the bacterial pathogen *Erwinia amylovora*. A broad, large-effect quantitative trait locus (QTL) for fire blight resistance has been reported on linkage group 3 of *Malus* ‘Robusta 5’. In this study we identified markers derived from putative fire blight resistance genes associated with the QTL by integrating further genetic mapping studies with bioinformatics analysis of transcript profiling data and genome sequence databases.

**Results:**

When several defined *E.amylovora* strains were used to inoculate three progenies from international breeding programs, all with ‘Robusta 5’ as a common parent, two distinct QTLs were detected on linkage group 3, where only one had previously been mapped. In the New Zealand ‘Malling 9’ X ‘Robusta 5’ population inoculated with *E. amylovora* ICMP11176, the proximal QTL co-located with SNP markers derived from a leucine-rich repeat, receptor-like protein ( *MxdRLP1*) and a closely linked class 3 peroxidase gene. While the QTL detected in the German ‘Idared’ X ‘Robusta 5’ population inoculated with *E. amylovora* strains Ea222_JKI or ICMP11176 was approximately 6 cM distal to this, directly below a SNP marker derived from a heat shock 90 family protein gene ( *HSP90*). In the US ‘Otawa3’ X ‘Robusta5’ population inoculated with *E. amylovora* strains Ea273 or E2002a, the position of the LOD score peak on linkage group 3 was dependent upon the pathogen strains used for inoculation. One of the five *MxdRLP1* alleles identified in fire blight resistant and susceptible cultivars was genetically associated with resistance and used to develop a high resolution melting PCR marker. A resistance QTL detected on linkage group 7 of the US population co-located with another HSP90 gene-family member and a WRKY transcription factor previously associated with fire blight resistance. However, this QTL was not observed in the New Zealand or German populations.

**Conclusions:**

The results suggest that the upper region of ‘Robusta 5’ linkage group 3 contains multiple genes contributing to fire blight resistance and that their contributions to resistance can vary depending upon pathogen virulence and other factors. Mapping markers derived from putative fire blight resistance genes has proved a useful aid in defining these QTLs and developing markers for marker-assisted breeding of fire blight resistance.

## Background

Fire blight, caused by the bacterial pathogen *E. amylovora* ( *Ea*), is a destructive disease of apple, pear and several other rosaceous species, infecting blossoms, fruit, vegetative shoots, woody tissues, and rootstock crowns. Although fire blight has been known as a disease problem for over 200 years [1], options for its management remain limited, and in the US, economic losses to fire blight and cost of control average over $100 million yearly [2-5]. Control is limited by three major problems: (i) most of the popular apple cultivars are either susceptible or highly susceptible to fire blight; (ii) many of the dwarfing rootstocks currently utilized are also highly susceptible to the disease; and (iii) the few chemical options available for control of scion infection, i.e. streptomycin, are further limited by the development of resistance to the antibiotic in areas where it is still registered for agricultural use [6-8].

*Ea* is native to eastern North America and was first reported in New Zealand (NZ) in 1919, and then Europe and the eastern Mediterranean in the 1950-60s [9,10]. There is greater genetic diversity among North American strains of *Ea* than either NZ or European strains [11,12]. Genetic studies suggest that there have been as many as four introductions of *Ea* into Europe, and due to their limited diversity they most likely were not the result of repeated introductions from North America [9]. It is presumed that *Ea* evolved as a pathogen of North American rosaceous species, such as *Crataegus* and *Sorbus*, and first came into contact with its current economically important hosts, *Malus* x *domestica* (apple) and *Pyrus communis* (pear), approximately 350 years ago during European colonization of North America.

In general, resistance to *Ea* in apple and pear is quantitative, but there is evidence that the high levels of resistance observed in *Malus* x *robusta* ‘Robusta 5’ (R5) and ‘Evereste’ could be monogenic with additional QTLs moderating the degree of susceptibility of sensitive seedlings [13,14]. Strains of *Ea* have been identified that are differentially virulent on specific resistant apple cultivars, including ‘Novole’, ‘Ottawa 523’, ‘Quinte’ and R5 [15-18]. However, to date no specific interactions between pathogen avirulence genes and host resistance genes have been defined.

Use of marker-assisted selection (MAS) for development of new apple cultivars with genetic resistance to diseases is feasible when resistance is conferred by major qualitative gene loci or is due to quantitative resistance conferred by several loci [3,19-22]. A QTL that explains 35-40% of the phenotypic variation for fire blight resistance has been identified on linkage group (LG) 7 of the scion cultivar ‘Fiesta’, for which a set of three markers spanning this QTL has been developed and validated [20,21,23]. Recently, Le Roux et al. [24] mapped a medium-effect QTL (16% of phenotypic variation) to ‘Florina’ LG10, while two other larger effect (>50%) QTLs from ‘Evereste’ and *M. floribunda* 821 were mapped to the lower end of LG12 [14,25], a region where resistances to scab and mildew have also been located [26,27]. R5 is a source of resistance in rootstock and scion breeding programs at PFR, JKI at Dresden-Pillnitz and USDA-ARS/Cornell University, Geneva, NY [28]. A large-effect QTL located by conventional interval mapping and explaining 67-83% of the phenotypic variation was reported on LG3 of the cultivar R5 [22]. This QTL has been confirmed in an ‘Idared’ x R5 (IxR5-DE) population in Germany [13,29], a ‘Malling 9’ x R5 (M9xR5-NZ) population in New Zealand [13] and an ‘Ottawa 3’ x R5 (O3xR5-US) population in the US [30]. In this study, we performed more detailed QTL analyses of fire blight resistance in all three families to delineate more closely the QTL region reported earlier [13].

Powerful genomics technologies are now available for both identification of putative resistance genes and marker development [31-33]. To develop robust markers to be used in MAS for breeding of fire blight resistant varieties, we identified possible fire blight resistance genes from EST and genomic databases, mapped derived genetic markers with respect to resistance QTLs and selected for MAS those markers closely correlated with the resistance [34-36]. Plant responses to pathogen invasion are highly complex interactions that can involve the transcription of 2000 to 3000 genes [37-40]. Starting with a published set of several hundred ESTs differentially expressed in *Malus* in response to *Ea* challenge [41,42], we used a combination of bioinformatics and inference from the scientific literature to identify putative fire blight resistance genes. Genetic markers were then developed from EST [43] or genomic sequence [33] and mapped in the three populations for which a QTL for fire blight resistance had previously been reported on LG3 [13,22,30]. In order to explore the possible functionality of two of these putative resistance genes in the fire blight resistance reactions, we allelotyped a leucine-rich repeat (LRR) receptor-like protein that mapped to the peak of the QTL for fire blight resistance in a range of fire blight resistant and susceptible germplasm accessions and performed a functional analysis on a class 3 peroxidase that mapped to the same location.

## Results and discussion

### Genetic analysis suggests differences in R5 accessions

In order to verify the identity of the three R5 accessions used in the three mapping populations, the accessions were genotyped with six SSR markers distributed over the upper 30 Mb of LG3 (Table1). The results demonstrate that while R5-DE and R5-NZ accessions are genotypically identical, there is a strong possibility that R5-US is not equivalent, as it differs in allelotype at marker NZmsCN943818. Our records cannot define the relationships among the R5 accessions; however, it is not unusual for clonal variants to arise in asexually propagated crops, or for errors to arise in records in germplasm collections [44-46].

**Table 1:** **Genetic comparison of three** ‘ **Robusta 5’ (R5) accessions from Germany (DE), New Zealand (NZ) and United States (US) using SSRs**

**Genetic marker**	**Physical Position (Mb)**^**1**^	**R5-NZ Allele sizes (bp)**^2^	**R5-DE Allele sizes (bp)**^2^	**R5-US Allele sizes (bp)**^2^
NZmsMdMYB12	0.97	170, 197	170, 197	170, 197
CH03e03	1.03	208, 230	208, 230	208, 230
NZmsMDC007176.537	1.81	176, 188	176, 188	176, 188
NZmsMDC018101.293	3.87	194	194	194
CH03g07	7.16	147, 165	147, 165	147, 165
NZmsCN943818	29.78	207, 227	207, 227	209, 211

Simple sequence repeat allele sizes in base pairs (bp) for markers mapping near the fire blight resistance QTL on Linkage Group 3 of apple.

^1^ The physical position of the markers is based upon the whole genome sequence of ‘Golden Delicious’ [33] with 200kbp gaps inserted between scaffolds [104].

^2^ Analyses were performed in duplicate. The alleles are not corrected to their true size.

### Quantification of resistance phenotype was improved by logit transformation of percent cumulative necrotic lesion length

**Figure 1:**
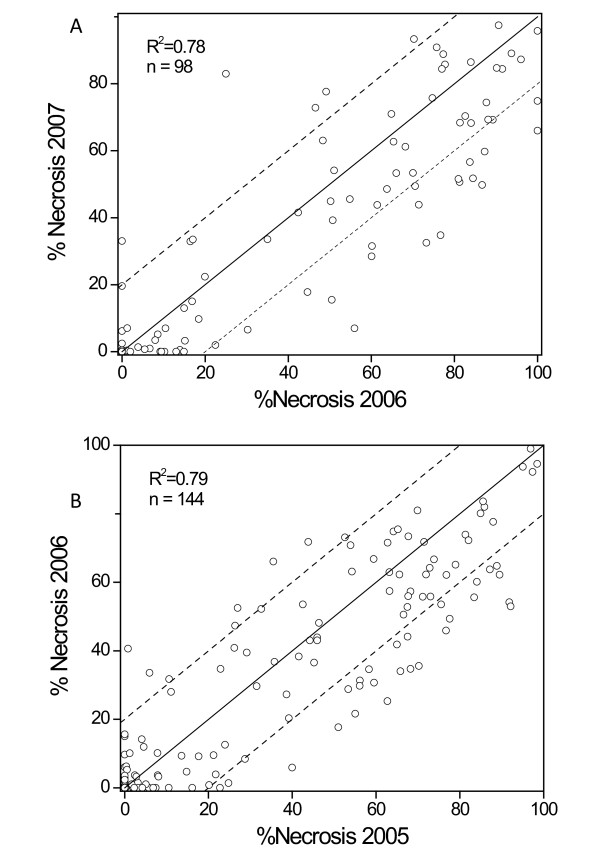
**Scatter plots of fire blight severity.** Scatter plot of percentage of the current season’s shoot length that became necrotic following inoculation with *E. amylovora* (% SLN) evaluated in A) 2006 and 2007 on 83 progeny of ‘Malling 9’ x ‘Robusta 5’-NZ, and B) 2005 and 2006 on 132 progeny of ‘Idared’ x ‘Robusta 5’-DE. The R^2^ value is for the linear line of best fit, and not for the 1:1 line as shown in the figure. Dashed lines indicate boundary for more than 20% difference between years.

The disease phenotype of all three progeny was defined by the percent cumulative necrotic lesion length of current season’s shoot length following inoculation with *Ea* (%SLN). Scatter plots of the %SLN data assessed over two years for M9xR5-NZ and IxR5-DE families inoculated with strain ICMP11176 and Ea222_JKI (Table2), respectively, showed no clear evidence of an annual effect on the phenotype (Figure1). When repeated measurements of phenotype are available for individuals in a mapping family, an estimate of repeatability can indicate whether there is a genetic basis for the trait response. In particular, repeatability gives an upper bound on heritability of the trait, so that low repeatability estimates are useful pointers to lack of a genetic influence. In this study, repeatability estimates calculated by the correlation coefficient were consistent and high (*r* = 0.89, with p < 0.001). Hence, mean %SLN was simply calculated by aggregating data over the two seasons for the R5-NZ and R5-DE populations. When the O3xR5-US family was previously inoculated with strains Ea273 and E2002a (Table2), there was a significant correlation between the disease phenotypes obtained with each strain. However, several progenies within the O3xR5-US family exhibited differential response to the two strains [30].

**Table 2:** **Strains of*Erwinia amylovora*used in this study**

**Strain**	**Geographic Origin**	**Host of Origin**	**Originally Isolated By**	**References**
E2002a^1^	Ontario, Canada	*M. X domestica*‘Jonathan’	W.G. Bonn	[15,17,106]
Ea222_JKI^2^	Havlickuv Brod, Czech Republic	*Cotoneaster* sp.	B. Kokoskova	[107]
Ea273	New York State, US	*M. X domestica ‘R.I. Greening’*	S.V. Beer	[15,17,108]
ICMP11176	Hawke’s Bay, NZ	*M. X domestica*‘Gala’	R.G. Clark (donor)	[87]

^1^ Also referred to as Ea265 [15,17].

^2^ Distinct from strain Ea222 previously cited by Norelli et al. [109], also referred to as Ea222 [107], original Czech designation was 50/92.

**Figure 2:**
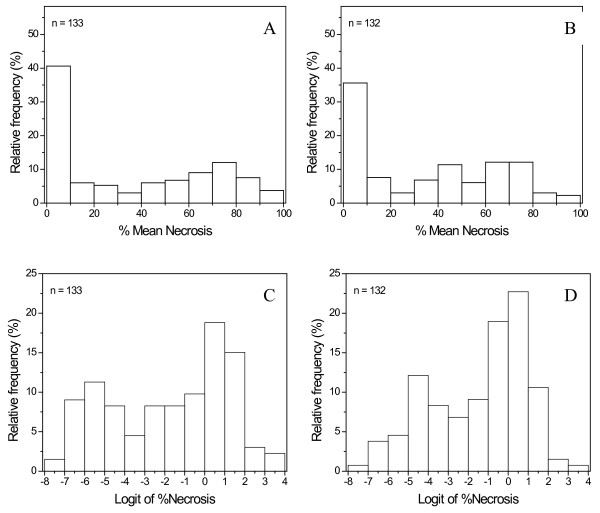
**Distribution of fire blight severity in ‘Robusta 5’ populations from New Zealand and Germany.** Histograms of fire blight mean % cumulative necrosis of current season’s shoot length over two seasons, presented as a percentage (top, A and B) and on the logit transformed scale (bottom, C and D), for the progeny of A/C: ‘Malling 9’ x ‘Robusta 5’ NZ and B/D: ‘Idared’ x ‘Robusta 5’ DE.

Plots of %SLN distribution of plants in both the M9xR5-NZ and IxR5-DE families showed a spike at or near zero mean %SLN and contained 35-40% of observed individual plant responses, with the remainder in a much flatter distribution, peaking around 50% (Figure2A, 2B). The logit transformation of the data (ln(%SLN / (100 - %SLN)) made the phenotypic distribution appear more like a mixture, and possibly that of two normal distributions (Figure2C, 2D). Absence of symptoms could be due to either true resistance of the host plant to invading pathogen or escape from pathogen inoculation. While disease symptoms are observable, the escape rate is not. Because R5 is known to transfer a high level of resistance to its progeny and we observed many plants with a high %SLN in all populations, we assumed that the escape rate was low.

### Putative fire blight resistance genes were selected from EST data and mapped in ‘Robusta 5’

Thirty five putative fire blight resistance genes were selected for mapping, from two sources. Eight were from a set of ESTs selected by inference from early versions of the PFR database [43] on the basis of possible association with disease resistance in general (Gardiner, unpublished). Twenty-seven putative fire blight resistance genes were selected specifically for this study from a set of over 650 fire blight-associated ESTs previously identified by suppression subtractive hybridization (SSH) [41] and cDNAamplified fragment length polymorphism (AFLP) [42] (see Additional file 1: Putative fire blight resistance genes).

Initially, we identified 883 unique contigs (581 clusters and 302 singletons) or ‘fire blight unigenes’ from 5,395 apple ESTs identified from *Ea*-challenged leaves of moderately resistant ‘Red Delicious’ and highly resistant G.41 apple rootstock [47] (see Additional file 2: Fire Blight Unigenes). An additional 200,115 ESTs from presumably non-infected apple tissues were downloaded from GenBank and clustered into 34,982 unique contigs (23,870 clusters and 11,112 singletons) or ‘non-fire blight unigenes’. Bioinformatics analysis then identified SSH and cDNA-AFLP ESTs that: 1) appeared unique when compared with ‘non-fire blight unigenes’ (‘*Ea* unique’ in Additional file 1, 2)) were similar to ‘fire blight unigenes’, SSH or cDNA-AFLP ESTs (‘*Ea* common’ in Additional file 1 and 3)) were similar to *Arabidopsis* genes regulated in response to bacterial challenge or by systemic acquired resistance (‘ *At* bacterial’, ‘ *At* SSH avir’, ‘ *At* SSH vir’ or ‘ *At* SAR’ in Additional file 1). We then selected putative fire blight resistance genes using both results from bioinformatics analysis and inferences drawn from model species and the scientific literature.

**Figure 3:**
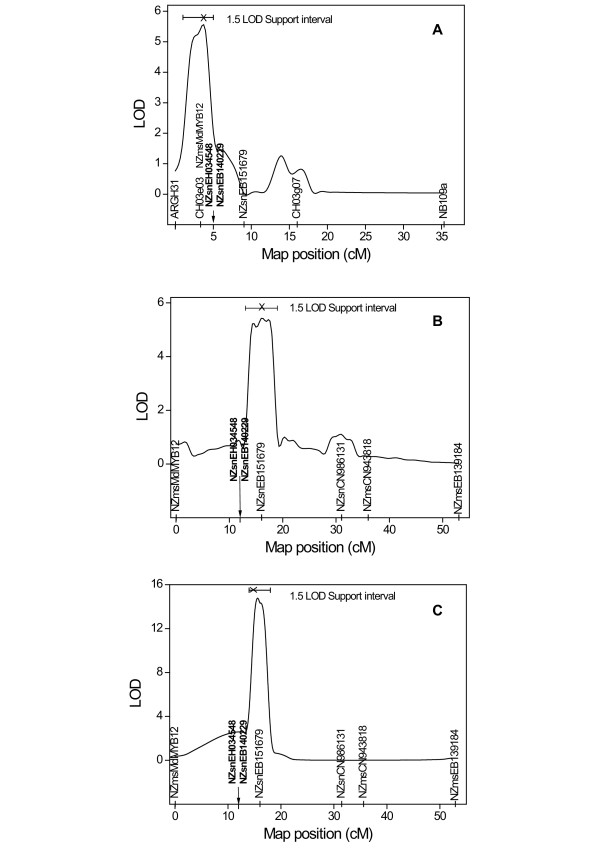
**QTLs on Linkage Group (LG) 3 of ‘Robusta 5’ for fire blight resistance identified in the ‘Malling 9’ x ‘Robusta 5’ New Zealand (M9xR5-NZ) and ‘Idared’ x ‘Robusta 5’ Germany (IxR5-DE) populations.** A)^*^ LOD score curves for Single QTL Composite interval mapping (CIM) in M9xR5-NZ following inoculation with *Erwinia amylovora* strain ICMP11176. B) LOD score curves for CIM in IxR5-DE following inoculation with *E. amylovora* strain ICMP11176 by the Multiple Imputation method using 128 imputed datasets and 1000 permutations to estimate LOD thresholds. C)^*^ LOD score curves for CIM in IxR5-DE following inoculation with *E. amylovora* strain Ea222_JKI. ^*^ In A and C markers outside a window length of 3 cM were used as co-factors; interval mapping method used was ‘Hayley-Knott’ regression; the 95% confidence limits estimated by 1.5-LOD drop-off method are shown at the top figure; and putative fire blight resistance genes NZsnEH034548 and NZsnEB140229 mapped to the same position (indicated by arrow) and were excluded from the analyses. The LOD thresholds at significance levels of 5% and 1% calculated from 1000 permutations were [1.8, 2.5], [1.6, 2.6] and [1.7, 2.3], respectively, for Figures 3A, 3B and 3C. Two alternative CIM methods (EM algorithm and Imputation) produced similar results.

**Figure 4:**
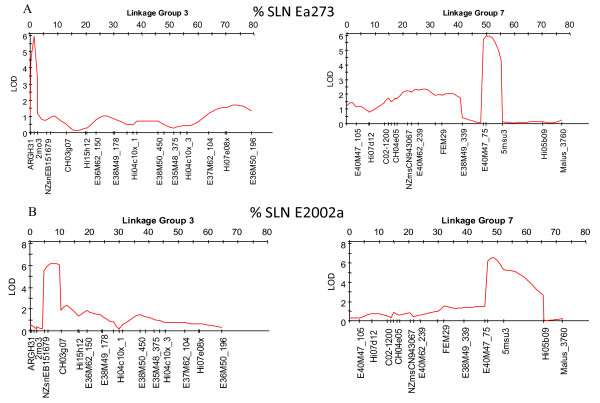
‘Robusta 5’ QTLs for fire blight resistance identified in the ‘Ottawa3’ x ‘Robusta 5’ United States (O3xR5-US) population on Linkage Groups 3 and 7. **‘Robusta 5’ QTLs for fire blight resistance identified in the ‘Ottawa3’ x ‘Robusta 5’ United States (O3xR5-US) population on Linkage Groups 3 and 7.** A) LOD score curves for Multiple QTL Mapping following inoculation with *Erwinia amylovora* strain Ea273 using MAPQTL 6 (Kyazma, Wageningen, NL) MQM mapping option. B) LOD score curves of Multiple QTL Mapping following inoculation with *Erwinia amylovora* strain E2002a using MAPQTL 6 (Kyazma, Wageningen, NL) MQM mapping option.

Eighteen markers derived from putative resistance genes were first located to bins in linkage groups using the M9xR5-NZ [48] or RGxA689 bin mapping sets [49], and then genetically mapped to linkage groups using the respective full populations (Table3). When the ‘Golden Delicious’ whole genome sequence (WGS) became accessible, these positions were checked against physical positions assigned within the WGS [33] (see Additional file 1, worksheet A). Six markers derived from putative resistance genes were bin-mapped, using one or other of the bin mapping sets (see Additional file 1, worksheet B), but not mapped in a full population. Eighteen further markers derived from putative resistance genes not able to be bin mapped (see Additional file 1, worksheet C), were physically mapped based upon their positions in the WGS. GenBank EH034548-, EB140229- and EB151679-derived markers were genetically mapped to the upper half of LG3 in M9xR5-NZ (Figure3) and were subsequently mapped in the IxR5-DE (22, Figure3). Of these three putative fire blight resistance genes, only EB151679 was mapped in the O3xR5-US [30] population (Figure4).

**Table 3:** **Distribution of putative fire blight resistance genes among*Malus*genetic linkage groups**

**Linkage Group**	**# SSRs**	**# SNPs**	**# WGS**
LG1	1	1	4
LG2	1	1	0
LG3	0	4	0
LG5	2	1	0
LG6	1	0	0
LG7	3	1	5
LG9	0	0	1
LG10	1	3	2
LG11	0	1	0
LG12	0	0	1
LG13	0	1	3
LG14	0	0	1
LG16	0	0	1
LG17	0	1	1

Thirty-six putative fire blight resistance genes were genetically mapped on *Malus* genetic linkage groups (LGs) as simple sequence repeat (SSR) and single nucleotide polymorphism (SNP) markers, or physically located within the ‘Golden Delicious’ whole genome sequence (WGS). See Additional file 1: Putative fire blight resistance genes for details of EST and marker description, criteria used for putative fire blight resistance gene selection, genetic and physical location of the markers within LG.

### Evidence for two QTLs for resistance to fire blight at the top of Linkage Group 3

QTL discovery for resistance to fire blight was conducted in three independent populations, i.e. M9xR5-NZ, IxR5-DE and O3xR5-US comprising 133, 132 and 186 phenotyped plants, respectively, of which 98.1%, 94.1% and 90.3%, respectively, were genotyped. Due to quarantine restraints on the exchange of plant material and *Ea* strains, the populations were separately inoculated with two different strains of *Ea* (Figures 3 and 4). As conventional interval mapping [50] in the presence of a moderate to large QTL within a relatively small region is likely to have low precision in positioning the QTL, we employed Composite Interval Mapping (CIM) or Multiple QTL Mapping (MQM) [51,52] for QTL identification. Both CIM and MQM adjust the interval map for linked markers by treating markers outside a specified window as co-variates and can increase the precision of QTL positions, as well as reduce errors due to the detection of ghost QTLs and failure to detect true ones. The positions of markers derived from putative resistance genes were lastly evaluated for possible correlation of their location with that of QTLs for resistance. In the case of M9xR5-NZ and IxR5-DE populations, QTL mapping was initially conducted without the NZsnEH034548- and NZsnEB140229-derived markers, in order to test how well the position of any putative QTL detected by mapping agreed with the estimated position of markers derived from putative resistance genes.

CIM analysis of the M9xR5-NZ and IxR5-DE populations following inoculation with *Ea* strain ICMP11176, enabled detection of apparently distinct QTLs for resistance to fire blight on LG3 of R5-NZ and R5-DE (Figure3A3B respectively). The LOD curves exhibited strong peaks and placed a single QTL at ~3 (LOD = 5.5) and ~16 (LOD = 5.4) cM on LG3 of R5 in M9xR5-NZ/ICMP (Figure3A) and IxR5-DE/ ICMP (Figure3B), respectively. Estimates of 95% confidence interval (CI) of the putative QTL positions determined by the 1.5 LOD drop-off method were 1–5 cM for M9xR5-NZ/ICMP and 13–19 cM for IxR5-DE/ICMP. The QTLs explained an estimated 17% of the variation in logit (%SLN) in both M9xR5-NZ/ICMP and IxR5-DE/ICMP. The peak of the fire blight resistance QTL on LG3 for M9xR5-NZ/ICMP (Figure3A) was more precisely defined than in Peil et al. [13] and was positioned over CH03e03 and NZmsMdMYB12. This QTL co-localized with markers NZsnEH034548 derived from a class 3 secretory peroxidase (GenBank:EH034548) and a LRR receptor-like protein (GenBank:EB140229) [41]. However, the QTL for IxR5-DE inoculated with the same strain mapped downstream from these markers and co-localized with NZsnEB151679-derived marker, indicating that there might be distinct QTLs in the two populations. The reason for such a difference is unclear, as R5-NZ and R5-DE had been shown to be genetically identical (Table1), the populations had been inoculated with the same strain of *Ea* and phenotyped using identical criteria. We speculated that this difference might be related to the genetic or physical environment of the two populations, which had different female parents and were located on opposite sides of the world, or be due to a statistical artifact arising from the limitations of mapping in bi-parental populations of small size, with low marker density.

When IxR5-DE was inoculated with a second strain of *Ea*, Ea222_JKI, a single QTL was located at ~15 cM of LG3 of R5-DE with a LOD score of 14 and 95% CI of 13–19 cM (Figure3C). The peak of this QTL was in the same position as when the inoculation was with ICMP11176 (Figure3B) and it co-localized with NZsnEB151679-derived marker.

To investigate whether the QTLs mapped were masking other additional QTLs, we used the additional QTL mapping (addqtl) function of R/qtl. This involved calculating LOD scores for each position for a two-QTL additive model, one of which is the QTL fixed at the position at which it has been already detected. When the NZsnEH034548- and NZsnEB140229-derived markers were included in the analysis, an additional QTL with LOD = 7 was detected at 13 cM on LG3 of IxR5-DE/Ea222, peaking close to these markers (results not shown). However, when the analysis was run without the markers, the results failed to support this finding, indicating the detection of an additional QTL may have been due to a statistical artifact. Because of the proximity of the additional QTL to the one already detected by CIM, conclusive proof of the existence of these two QTLs on LG3 of IxR5-DE/Ea222 would require validation in an expanded IxR5-DE population. Although the addqtl analysis was performed for M9xR5-NZ/ICMP, there was no evidence of additional QTLs (results not shown). No fire blight resistance QTLs were detected outside LG3 in either the M9xR5-NZ or IxR5-DE population in this, or in the previous study by Peil et al. [13].

Extension of the study to include the O3xR5-US population inoculated with two additional strains of *Ea*, Ea273 and E2002a (O3xR5-US/Ea273 and O3xR5-US/E2002a, respectively), also indicated that there were two distinctly positioned QTLs on LG3 (Figure4). The QTL on LG3 of R5 in O3xR5-US/Ea273 (Figure4A) was distinctly positioned with respect to the QTL detected in O3xR5-US/E2002a (Figure4B). The O3xR5-US/Ea273 QTL peaks at marker ARGH31 at 1.5 cM and its lower boundary was delineated by marker 2mo3 (Figure4A), while the upper boundary of the O3xR5-US/E2002a QTL peaks between markers NZsnEB151679 and CH03g07 at 8 cM (Figure4B). Both QTLs had LOD scores of at least 6 and explained genetic variance was 25% and 36%, respectively. Although the genetic maps for O3xR5-US (Figure4) and M9xR5-NZ/ IxR5-DE (Figure3) populations lack markers in common on LG3 distal to NZsnEB151679, it can be inferred from analysis of WGS [33] that marker sequence from the top of the LG3 is: NZMdMYB12, ARGH31, NZsnEH034548, 2mo3, NZsnEB140229, NZsnEB151679. In this case, the M9xR5-NZ/ICMP (Figure3A) and O3xR5-US/Ea273 (Figure4A) QTLs on LG3 would be separated from the IxR5-DE/ICMP (Figure3B), IxR5-DE/Ea222 (Figure3C), and O3xR5-US/E2002a (Figure4B) QTLs by the 2mo3 marker. The difference in location of the LG3 QTLs in the O3xR5-US/Ea273 and O3xR5-US/E2002a QTLs is almost certainly strain specific, since the resistance evaluation of the O3xR5-US population with strains Ea273 and E2002a was conducted in the same environment [30], not split between NZ and DE as for M9xR5-NZ/ICMP and IxR5-DE/ICMP populations. The North American strains Ea273 and E2002a are known to differ in their virulence to several fire blight resistant cultivars, including R5 [15,16]. The apparent differences in QTL peaks following inoculation of R5 populations in NZ and DE with ICMP11176 and Ea222_JKI (Figure3A3C) could also be due to a strain-specific response. However, other influences cannot be ruled out in this case, as the populations had different female parents and were evaluated in different environments. Nonetheless, taken together our results strongly suggest that the position of the QTL at the upper part of LG3 can be affected by virulence of the *Ea* strain and other unknown factors that warrant further investigation. Although environment, female parent, marker density, mapping population, and mapping algorithms differed in the O3xR5-US versus M9xR5-NZ and IxR5-DE QTL analyses, the two QTLs detected in the O3xR5-US and the M9xR5-NZ/ IxR5-DE populations appeared to be in the equivalent positions. This suggests that the shifting position of the detected QTLs on LG3 was not an artifact of any specific analysis.

Each of the three putative fire blight resistance genes that mapped to the upper part of LG3 of the R5 accessions in this study co-located with more than one QTL for the resistance. NZsnEH034548 (*MxdPrx8*)- and NZsnEB140229 (− *MxdRLP1*)-derived markers were associated with QTLs mapped in M9xR5-NZ/ICMP using CIM, as well as the possible additional QTL for resistance in IxR5-DE/Ea222 identified using addqtl. As the CIM analysis in the M9xR5-NZ population was performed excluding the data for NZsnEH034548- and NZsnEB140229-derived markers, the close positioning of a QTL for fire blight resistance to these putative resistance gene markers could be considered as strong evidence for their association with fire blight resistance. NZsnEB151679 (HSP90)-derived marker was located within the IxR5-DE/ICMP and IxR5-DE/Ea222 QTLs, as well as the O3xR5-US/E2002a QTL. These observations of co-location of QTL peaks on LG3 with putative fire blight resistance genes suggest that multiple genes within the upper part of LG3 contribute to fire blight resistance and that their respective contributions to resistance can vary under the influence of multiple biological factors.

Furthermore, analysis of the O3xR5-US population enabled identification of a QTL for resistance to fire blight on LG7 of R5. The peaks of the strong (LOD > 6) QTLs on LG7 were mapped between markers E40M47-75 and 5Msu3 at about 48 cM, in the O3xR5-US/Ea273 and O3xR5-US/E2002a populations. Analysis of the WGS [33] indicates that the O3xR5-US/Ea273 QTL is located higher on LG7 than the FB7 QTL from ‘Fiesta’ [21] (results not shown). However, the O3xR5-US/E2002a QTL extends further down the LG than the O3xR5-US/Ea273 QTL and hence would overlap with the FB7 QTL (Figure4).

### RT-qPCR analysis suggests a functional role of *MxdPrx8* (GenBank:EH034548) in fire blight resistance

Previous transcript profiling experiments identified five *Malus* peroxidase ESTs that are differentially regulated in response to fire blight challenge in susceptible ‘Gale Gala’ [41]. EST GenBank:EH009551 had been annotated as a Class 1 cytosolic ascorbate peroxidise (APX). The other four ESTs had been annotated as Class 3 secretory peroxidases (Prx) and were selected in this study as putative fire blight resistance genes (see Additional file 1: Putative fire blight resistance genes). Based upon initial BLASTn comparison to *Malus* EST assemblies [53,54] and subsequent mapping, these Prx ESTs were found to represent two distinct gene family members. GenBank:EH034548 (*MxdPrx8*)-derived marker co-located with the upper of the two QTLs for fire blight resistance on LG3 of R5 shown in Figures 3 and 4 (NZsnEH034548, see Additional file 1)*.* ESTs GenBank:EH034563, EH009531 and EH034487 ( *MxdPrx9*)-derived marker mapped to LG10 above the position of a QTL mapped in ‘Florina’ [24] (NZsnEB133738, see Additional file 1).

**Figure 5:**
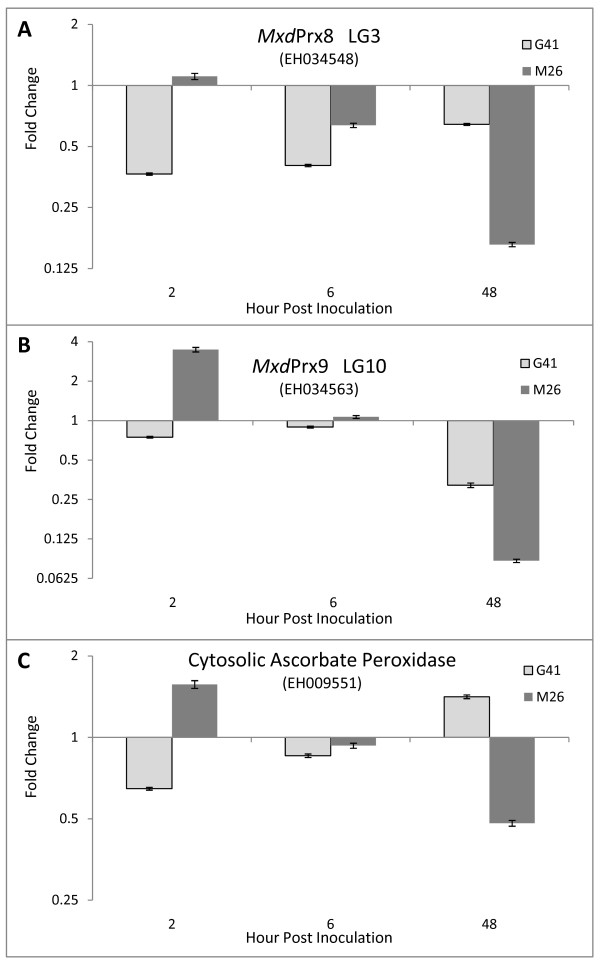
**Relative transcript abundance of three peroxidase genes in ‘Geneva 41’ and ‘Malling 26’ apple rootstocks.** Fold change in transcript abundance of three peroxidase genes in fire blight resistant ‘Geneva 41’ (light gray) and susceptible ‘Malling 26’ (dark gray) *Malus* rootstocks following inoculation with *Erwinia amylovora.* A: *MxdPrx8*, a class 3 peroxidase that co-located with a QTL for fire blight resistance on LG3 of ‘Robusta 5’-NZ; B: *MxdPrx9*, a class 3 peroxidase that mapped to Linkage Group 10; and C: a class 1 cytosolic ascorbate peroxidise. Transcript abundance was determined by RT-qPCR, is expressed as fold change in comparison to mock challenged tissue of the same genotype sampled at the same hpi and is represented on a log2 scale to equalize magnitude of induced and repressed gene expression; a fold change of 1 indicate no difference from mock-inoculated (reference) whereas values less than 1 indicate repression of gene expression. Transcript abundance was normalized to an elongation factor 1 internal control and fold change calculated by the 2^-ΔΔCt^ method; error bars are the ΔΔCt standard deviation calculated from the technical replicates [3] of all 3 biological replicates (total of 9) [103]. EST sequence used for PCR primer design is indicated in brackets (GenBank accession number).

To determine the effect of fire blight challenge on transcript abundance of these peroxidase genes, leaf tissue was mock or *Ea* inoculated, leaf samples harvested 2, 6 and 48 h post inoculation (hpi), and RT-qPCR performed on extracted RNA. The general temporal pattern of expression of the peroxidase genes in response to pathogen challenge in the susceptible ‘M.26’ cultivar was an initial induction of gene expression at 2 hpi, followed by down-regulation leading to an eventual repression of gene expression by 48 hpi (Figure5). In contrast, the general response in the resistant ‘G.41’ was a rapid repression of gene expression by 2 hpi that was then relaxed over time (Figure5A-5C). In summary, when comparing transcript abundance in the resistant versus susceptible cultivar: 1) at 2 hpi there was significantly less expression of *MxdPrx8*, *MxdPrx9* and APX in ‘G.41’ than ‘M.26’ (all P = <0.0001); 2) at 6 hpi there was significantly less expression of only *MxdPrx8* (P = 0.01) in ‘G.41’; and 3) at 48 hpi there was significantly less expression of all three genes in ‘M.26’ than in ‘G.41’ (all P = <0.0001).

Class 3 peroxidases (E.C. 1.11.1.7) form large multigene families [55-57] and are ascribed a wide variety of functional roles in plant biology, including cross-linking of cell wall constituents, lignin polymerization, catabolism of auxin and the formation of reactive oxygen species [58]. They play a prominent role in defense reactions against a wide range of pathogens, including bacteria [59,60]. Transgenic *Arabidopsis* plants expressing an anti-sense cDNA encoding a class 3 peroxidase from French bean (FBP1) exhibit an impaired oxidative burst and greater susceptibility than wild-type plants to both fungal and bacterial pathogens [59]. Transcript profiling and RT-qPCR analysis showed that the transgenic anti-sense FBP1 *Arabidopsis* plants have reduced levels of a specific peroxidase-encoding mRNAs corresponding to At3g49120, that encodes a class 3 peroxidase with a high degree of homology to FBP1 [39] and also to *MxdPrx8* (tBLASTx 3e-115, *At*Prx34). Although it appears contradictory that in *Arabidopsis* down-regulation of *At*Prx34 is associated with an increase of susceptibility, whereas in *Malus* down-regulation of *MxdPrx8* was associated with an increase of resistance (Figure5), the oxidative burst that usually is associated with resistance in most host-pathogen interactions normally occurs during a susceptible fire blight response [61,62]. Therefore, if down-regulation of *MxdPrx8* resulted in an impaired oxidative burst in *Malus*, it is possible that this could result in an increase in fire blight resistance. Among the three peroxidase genes evaluated by RT-qPCR, *MxdPrx8* demonstrated the greater level of down-regulation in the resistant cultivar ‘G.41’ early in the host-pathogen response. However, both class 3 peroxidases and a class 1 peroxidase were down-regulated in fire blight resistant ‘G.41’ in comparison to susceptible ‘M.26’ (Figure5), suggesting that peroxidase expression in these cultivars may be the result of resistance rather than a regulator of resistance. To address this question, susceptible ‘M.26’ has been transformed with DNA vectors designed to individually silence *MdxPrx8* and *MdxPrx9* so that the effects of down-regulating these genes on fire blight resistance can be determined.

### Bioinformatic analysis suggests EB140229 is a leucine-rich repeat, receptor-like protein (*MxdRLP1*)

**Figure 6:**
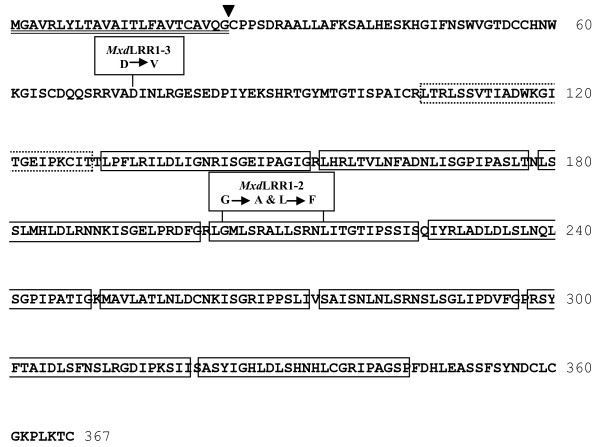
**Sequence and structure of*Malus*leucine-rich repeat family receptor-like polypeptide.** Predicted translation product of the *MxdRLP1-1* allele cloned from cultivars ‘Malling 26’, ‘Malling 27’ and ‘Robusta 5’. A predicted signal peptide cleavage site is indicated by an inverted arrowhead, and the signal peptide is double underlined. Nine consensus (LXXLXXLXLXXNXXαGXαPXXαG, where α represents any hydrophobic amino acid substitution [105]) LRR elements are boxed; the tenth degenerate element is boxed with a dotted line. The two mutated alleles isolated from cultivar ‘G.41’ are shown in boxes above the corresponding sequence.

The predicted DNA sequence for for EB140229 was derived from an EST consensus sequence 43, 59–60). The EST consensus sequence predicted an ATG start, 1,104 bp coding region and 3' untranslated region, and EB140229 aligned to the first 571 bp of the 1,543 bp consensus sequence. The sequence had significant similarity (BLASTx e-142, 72% amino acid sequence identity) to an *Arabidopsis thaliana* leucine-rich repeat (LRR) family protein (GenBAnk:NP188718) containing a LRR N-terminal domain (pfam08263) and a ribonuclease inhibitor-like subfamily LRR domain (cd00116,) consisting of a beta strand with a conserved amino acid pattern (LxxLxLxxN/CxL) and an alpha helix (Figure6).

**Figure 7:**
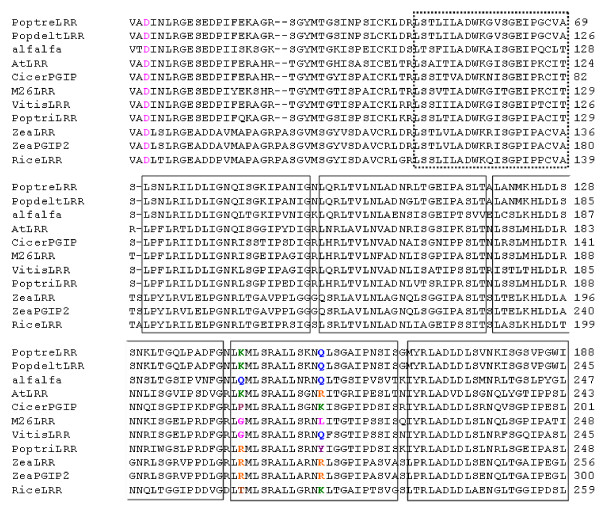
**Alignment of monocot and dicot leucine-rich repeats (LRR) homologues of the allele*MxdRLP1.*** Only the regions where amino acid replacements were detected in the apple LRRs are shown in the alignment. Numbers of amino acids from the presumed translation start methionine are listed to the right of the sequence. Amino acid substitutions at indicated loci are shown color-coded to highlight conservation or variation. Rectangular boxes indicate the positions of consensus LRRs. A dashed rectangle indicates deviation from the LRR consensus sequence. AtLRR: *Arabidopsis thaliana* (GenBank:NP188718, locus tag AT5G20820) ribonuclease inhibitor-like (RIL) LRR subfamily; PoptreLRR: *Populus tremula* (GenBank:ACE97248) RIL LRR subfamily; PopdeltLRR: *P. deltoides* (GenBank:ABS18952) RIL LRR subfamily; PoptriLRR: *P. trichocarpa* (GenBank:ABK92966) LRR; Alfalfa: *Medicago truncatula* (GenBank:ACJ85058); VitisLRR: *Vitis vinifera* (GenBank:CAO21943) RIL LRR subfamily; CicerPGIP: *Cicer arietinum* (GenBank:CAD56505) polygalacturonase inhibitor-like protein (PILP); ZeaLRR: *Zea mays* (GenBank:ACF88180) RIL LRR subfamily; ZeaPGIP2: *Z. mays* (GenBank:NP001150670) PILP; RiceLRR: *Oryza sativa* (GenBank:NP001062185); *Mxd*RLP1-1: allele (GenBank:XX000002) found in the fire blight susceptible *Malus* x *domestica* cultivars ‘Malling 26’, ‘Malling 27’ and fire blight resistant ‘Robusta 5’.

Three distinct classes of resistance proteins share the common LRR motif [63,64]. They include the multi-domain NB-LRRs (TIR-NB-LRR/CC-NB-LRR) [65], the receptor-like kinases (RLKs) containing an extracellular LRR, single-pass transmembrane domain and a cytoplasmic kinase domain [66,67], and the receptor-like proteins (RLPs) that differ from RLKs in that they lack the cytoplasmic kinase domain and only have a short cytoplasmic tail [68,69]. The derived *MxdRLP1* polypeptide was analyzed for the presence of a signal peptide directing sub-cellular localization. PSORT [70], PSIPRED [71] and Polyphobius [72] all predicted a signal peptide with a cleavage point between threonine (T)19 and cysteine (C)20, or between G24 and C25 (Figures 6 and 7). PSORT and Polyphobius also predicted that *MxdRLP1* would be extracellular. TMpred [73] and MEMSAT3 [74] were used to predict transmembrane regions for *MxdRLP1* but, neither predicted any transmembrane helices with confidence (not shown). Thus, the polypeptide associated with EST EB140229 is predicted to encode a LRR receptor-like protein ( *MxdRLP1)* that possesses a signal peptide and is likely to localize to the outside of the cell. It also contains the conserved 24 AA consensus motif (LxxLxxLxxLxLxxNxLxGxIPxx) associated with extracellular plant resistance gene LRRs [75] in the first, third and fifth LRR motif (allowing for leucine (L) - isoleucine (I) substitutions, Figure7), and 90% conservation of the motif in four other LRR repeats, which supports a possible role for this protein in disease resistance. In contrast to NB-LRRs and RLKs, very little is known about RLP signaling, although several important R genes are of the RLP type. These include *Cf-9* and *Ve* in tomato conferring resistance against *Cladosporium fulvum* and *Verticillium*, respectively, and *HcrVf-2* (aka *Vfa2*) in *Malus* against *Venturia inaequalis*[76,77]*.*

### Genetic analysis of *MxdRLP1* results in high resolution melting marker for fire blight resistance

DNA clones of *MxdRLP1* were obtained by high fidelity PCR amplification of genomic DNA template from R5 accessions, fire blight resistant R5 progeny and susceptible ‘M.9’, ‘M.26’, ‘M.27’ rootstocks using DNA primers designed from the EST consensus sequence. Alignment of independent *MxdRLP1* PCR clones identified five *MxdRLP1* alleles that were distinguished from each other by 16 SNPs, including four SNPs coding for amino acid substitutions (Table4). The SNP patterns identifying the five alleles occurred in multiple clones of each allele from each cultivar (Table4). SNPs occurring in a single sequence read or in the forward and reverse sequence read of an individual clone that did not align with SNPs in other clones were assumed to be either sequencing or PCR amplification errors, respectively, and were not included in allele identification. Similarly to their *Arabidopsis* homologue (At3g20820), the *Malus* alleles contained no introns. The predicted amino acid (AA) sequence of alleles *MxdRLP1*-1 (GenBank: JN798165, JN798170, JN798171) and −5 (GenBank: JN798169) were identical to that predicted by the EST consensus sequence and were identified in both resistant and susceptible cultivars (Table4). Allele *MxdRLP1*-2 (GenBank: JN798166) was correlated with resistance to fire blight in that it was identified in resistant R5-DE, R5-US and R5-NZ, and resistant progeny ‘G.41’ (GenBank: JN798172) and AJ103, yet absent from susceptible cultivars ‘M.9’, ‘M.26’ and ‘M.27’. The DNA sequence of *MxdRLP1*-2 encoded two AA substitutions within a single LRR domain (Table4, Figures 6 and 7). *MxdRLP1*-3 (GenBank: JN798167), which was identified only among DNA clones of fire blight resistant ‘G.41’, encoded one AA substitution, aspartic acid (D) in position 74 to valine (V) (Table4, Figures 6 and 7). *MxdRLP1*-4 (GenBank: JN798168), which was identified only among DNA clones of ‘M.27’, also contained one AA substitution.

**Table 4:** **Alleles of the*Malus*leucine-rich repeat family receptor-like protein gene (*MxdRLP1*)**

**Base Pair**^**1**^	**EST consensus**^**2**^	***Mxd*RLP1-1**	***Mxd*RLP1-2**	***Mxd*RLP1-3**	***Mxd*RLP1-4**	***Mxd*RLP1-5**
126	A	A	**G**	A	A	A
135	T	T	T	**C**	T	T
221	A	A	A	T*	A	A
405	C	C	**A**	C	C	C
438	C	C	C	C	C	**A**
451	G	G	G	G	A*	G
456	C	C	**A**	C	C	C
465	A	A	**T**	A	A	A
611	G	G	C*	G	G	G
645	G	G	T*	G	G	G
651	C	C	C	C	**T**	C
756	G	G	G	G	G	**T**
897	**G**	C	C	C	C	C
969	A	A	**G**	**G**	A	A
1053	G	G	G	G	G	A
1062	**C**	T	T	T	T	**C**
**Found in**^**3**^**:**		M.26 (1)	AJ103 (5)	G.41 (3)	M.27 (3)	M.9 (10)
		M.27 (7)	G.41 (3)			AJ103 (3)
		R5-DE (3)	R5-DE (5)			
		R5-NZ (8)	R5-NZ (4)			
		R5-US (4)	R5-US (3)			

Single nucleotide polymorphisms (SNPs, in bold type) detected in five alleles of putative fire blight resistance gene *MxdRLP1* that was identified from EST GenBank:EB140229, co-locates with the fire blight resistance QTL on LG3 and is homologous to *Arabidopsis thaliana* leucine-rich repeat family protein gene *At3g20820* (Genebank:NP188718). Asterisk (*) indicates SNPs resulting in amino acid substitutions.

^1^Base pair numbered from adenine of presumed ATG start codon of GenBank accessions JN798165-798172.

^2^Consensus sequence resulting from alignment of 1) Genome Database for the Rosaceae *Malus* EST assembly version 4 contig17331 [53], 2) TIGR *Malus* x *domestica* transcript assembly release 2 contig TA23938_3750 [54], and 3) the PFR bioinformatics database [43] sequence for *MxdRLP1*. The GDR and TIGR contigs were selected based upon BLASTN comparison with EST EB140229. ESTs within consensus sequence were derived from several different cultivars.

^3^ Alleles of *MxdRLP1* were identified from sequence alignment of multiple DNA clones obtained from fire blight resistant AJ103 (M9xR5-NZ progeny), ‘Geneva 41’ (G.41), ‘Robusta5’-DE (R5-DE), ‘Robusta 5’-NZ (R5-NZ), and ‘Robusta 5’-US (R5-US), and susceptible ‘Malling 9’ (M.9), ‘Malling 26’ (M.26), and ‘Malling 27’ (M.7). Number of DNA clones obtained for each allele is in parentheses following cultivar name.

Alignment of the *MxdRLP1* AA sequences with apparent homologues from a variety of phylogenetically representative plants indicated the *MxdRLP1*-2 and *MxdRLP1*-4 substitutions are in positions that appear to allow for considerable flexibility in AA substitution, whereas substitution in the *MxdRLP1*-3 pre-protein is in a highly conserved position (Figure7). A program designed to estimate the stability of a polypeptide with a single AA substitution, I-Mutant [78], predicted the *MxdRLP1*-3 allele to be significantly less stable than the *MxdRLP1*-1 allele, whereas, the *MxdRLP1*-2 allele appeared to be essentially equivalent to the *MxdRLP1*-1 allele in predicted stability (Table5). To evaluate the effect of these alleles on resistance, ‘M.26’ has been transformed with DNA vectors designed to over-express *MxdRLP1*-1,-2 and −3. Although the functional role of *MxdRLP1* requires further investigation, because allele *MxdRLP1*-2 is correlated with resistance it provides a useful marker for current MAS.

**Table 5:** Protein structural predictions for the three apple LRRs based on their primary sequences

**Apple cultivar**	**LRR allele**	**Signal peptide^1^**	**Location^2^**	**Predicted stability (ΔΔG)^3^**
‘M.26’, ‘M.27’, R5-US, R5-NZ	*MxdRLP1*-1	1-24	Outside cell	NA
R5-US, ‘G.41’, R5-NZ, AJ103	*MxdRLP1*-2	1-24	Outside cell	−0.95 ^4^; -0.05 ^5^ Kcal/mol
‘G.41’	*MxdRLP1*-3	1-24	Outside cell	−1.98 Kcal/mol

^1^Predictions were based on analysis by PSORT [70], PSPIRED [71] and Polyphobius [72].

^2^Prediction was based on PSORT [70] and Polyphobius [72].

^3^Predictions were based on analysis by I-Mutant2.0 [78]. Standard error for predictions based on protein sequence is 1.45.

^4^Allele *MxdRLP1*-2 using only the Gly to Ala substitution was used to perform prediction analysis, since the I-Mutant2.0 program will only analyze one amino acid substitution at a time from a single polypeptide.

^5^Allele *MxdRLP1*-2 using only the Leu to Phe substitution was used to perform prediction analysis.

The markers associated with the R5 resistance are all SNP markers, which are readily usable for selection of genetically elite seedlings in breeding populations. We find that analysis of SNPs by HRM using a LightCycler® 480 [49] to be a speedy and efficient method for routine medium-throughput MAS. Amplicons developed from PCR primers designed around the SNPs at bp 456 and bp 465 of *MxdRLP1*-2 (Table4) (forward primer: 5’ TCCGAATCCTTGACCTCATC 3’, reverse: 5’ CGAAGTTGAGAACCGTGAGA 3’) exhibited distinctive melting curves for a replicated series of fire blight resistant and susceptible accessions, when HRM analysis was performed on a LightCycler® 480 instrument [49]. The melting curves for samples from fire blight resistant R5-US, R5-DE, and R5-NZ and ‘G.41’ were clearly distinguishable from for fire blight susceptible ‘M.26’ and ‘M.9’ and both of these groupings from resistant progeny AJ103 (not shown). Direct sequencing of PCR products showed that the sequences of R5-US, R5-DE, R5-NZ, ‘G.41’ and AJ103 were consistent with possession of an allele derived from a common origin and differed from ‘M.9’ and ‘M.26’ at positions bp 456 and bp 465. The new marker mapped identically to EB140229 in the M.9xR5-NZ population (results not shown).

### Several putative resistance genes are physically located under the QTL on LG7 of ‘Robusta 5’

To identify putative fire blight resistance genes within the QTL identified on LG7 of R5-US, we scanned predicted protein coding sequences (MDPs) from the ‘Golden Delicious’ WGS both for genes differentially regulated following inoculation with *Ea*[41,42] and for possible pathogen receptors. Among the 496 MDPs identified between markers 5msu3 (51.6 cM, 21.85 Mbp) and Hi05b09 (64.9 cM, 25.98 Mbp), 8.3% (41 MDPs, see Additional file 3: Predicted coding regions within fire blight resistance QTL on Linkage Group 7 of ‘Robusta 5’ (R5-US) accession) showed significant similarity to ESTs differentially expressed in response to Ea challenge (fb-ESTs) that were previously identified by cDNA-AFLP [42], SSH [41] and microarray analysis [79], as well as the fire blight unigenes described in this work (see Additional file 2: Fire Blight Unigenes and Materials and Methods). In addition, 23 possible receptor proteins were also identified within this region, including 16 NB-LRR and seven receptor-like kinases (see Additional file 3).

A striking similarity between the fire blight resistance QTLs on LG3 and LG7 of R5-US is the presence of a HSP90 gene-family member on LG7 (MDP0000303430), that is similar to the HSP90 putative fire blight resistance gene associated with the lower fire blight resistance QTL on LG3 (marker NZsnEB151679). A protein complex of HSP90, SGT1 (for suppressor of G2 allele of skp1) and RAR1 (for required for *Mla12* resistance) is known to be important in the regulation of NB-LRR proteins in plants and is essential for disease resistance triggered by a number of NB-LRR resistance proteins [80,81]. This includes RPS2- and RPM1-mediated resistance to the bacterial pathogen *Pseudomonas syringae* in *Arabidopsis,* where both resistance proteins are activated via degradation of *Arabidopsis* RIN4 (for RPM1 interacting protein 4) by *Pseudomonas* effector proteins AvrRpt2 and AvrRpm1/AvrB, respectively [82-84]. Interestingly, the LG7 QTL also contains homologues of the HSP90 co-chaperone SGT1b (MDP0000318031) and RPM1 resistance protein (MDP0000137113, MDP0000249156, MDP0000395902, MDP0000252913). The SGT1b homologue was previously identified as differentially regulated in response to *Ea* challenge by SSH analysis (GenBank: EH034494) [41]. HSP90, SGT1b and RAR1 can independently interact with one another via the N-terminal ATPase domain of HSP90, which is known to interact with both the CS domain of SGT1b and the CHORDI domain of RAR1 [84]. Three amino acids of the HSP90 ATP-binding domain known to be essential for RPM1-mediated resistance in *Arabidopsis*[82] are conserved in all three of the fire blight-associated *Malus* HSP90 homologues, MDP0000303430 (LG7), MDP0000607364 (LG3) and MDP0000265782 (LG11). There are no QTLs reported for fire blight resistance on LG11.

Other fire blight resistance candidate genes within the LG7 QTL of R5-US include a putative WRKY transcription factor (MDP0000767097) previously found to be up-regulated in highly resistant ‘G.41’ (GenBank: EX878972) [42] and ‘Evereste’ (GenBank: AY347836) following *Ea* challenge. This WRKY appears highly specific to fire blight resistance in that ESTs EX878972 and AY347836 have no other significant *Malus* EST BLASTn matches. Increased expression of EX878972 (aka 175-G.41-48I) in resistant ‘G.41’ and decreased expression in susceptible ‘M.26’ following *Ea* challenge was previously confirmed by RT-qPCR [42].

### RIN4 gene mapped near published QTL on Linkage Group 10

A medium effect QTL for resistance to fire blight has been located on LG10 of ‘Florina’ [24]. It was particularly interesting that CV629535 (RIN4-like protein), could be located on the WGS 4 Mb below the edge of the confidence limits of the ‘Florina’ QTL (see Additional file 1B), as RIN4 is believed to be a negative regulator of plant PAMP-triggered immunity to microbes [85,86].

## Conclusions

Integration of the genetic analysis of QTLs for fire blight resistance in R5 with bioinformatics analysis of EST and genome sequence databases enabled us to identify markers derived from putative fire blight resistance genes. We also demonstrated how careful consideration of quantification of a complex disease phenotype and increased marker density enabled the resolution of two distinct QTLs in the upper part of LG3, where only one had been previously mapped [22]. In the M9xR5-NZ and I xR5-DE populations, the peaks of LOD curves for fire blight resistance were located 5–6 cM apart in the region between the markers CH03e03 and CH03g07 (Figure3). The upper of these two QTLs co-localized with NZsnEH034548-derived marker for a class 3 secretory peroxidase (*MxdPrx8*) and NZsnEB151679-derived marker for a LRR receptor-like protein ( *MxdRLP1*), whereas the lower QTL co-localized with the NZsnEB151679-derived marker for a heat shock protein HSP90 candidate resistance gene. When the O3xR5-US population was inoculated with strains known to differ in their virulence to R5, two distinct strain-dependent QTLs were detected in locations corresponding to those detected for the two QTLs in the M9xR5-NZ and IxR5-DE populations (Figure4). RT-qPCR analysis found transcript abundance of class 3 secretory peroxidase candidate resistance gene GenBank:EH034548 (*MxdPrx8*) was regulated differently in fire blight resistant ‘G.41’ and susceptible ‘M.26’, supporting a functional role for marker NZsnEH034548, which co-located the upper of the two QTLs on LG3 of R5.

The results suggest that the upper part of LG3 contains multiple genes contributing to fire blight resistance and that their contributions to resistance can vary depending upon pathogen virulence and other undetermined factors. Further research to identify host gene by pathogen gene interactions within this region is warranted.

Five alleles of the putative LRR-RLP resistance gene (*MxdRLP1)* from LG3 were identified from both fire blight resistant and susceptible *Malus* cultivars. Allele *MxdRLP1-2* was found in R5 and its resistant progeny but not in susceptible cultivars. This allele contains two AA substitutions within one of the LRRs and was used to develop an HRM candidate gene marker for resistance.

A QTL for fire blight resistance that was detected on LG7 of R5-US was not detected in either the M9xR5-NZ or the IxR5-DE populations (Figure4). Analysis of the *Malus* whole genome sequence within the QTL on LG7 identified another gene-family member of the HSP90 putative fire blight resistance gene (marker NZsnEB151679) and a WRKY transcription factor previously associated with resistance to fire blight, both of which need further investigation.

While R5 has been predominantly used by breeders to confer fire blight and woolly apple aphid (*Er2*; 34) resistance in new dwarfing rootstocks, backcrossing of these resistances into new scion cultivars is now in progress, and F2 selections with improved size and fruit quality have been made in New Zealand. The R5 fire blight resistance will be pyramided with fire blight resistances from other genetic backgrounds to improve durability and enable long-term deployment of genetic resistance for fire blight control in apple production systems. Although the position of the LG3 QTL varied with strain, if a combination of markers designed for allelotypes of Prx/RLP and HSP90 associated with resistance is used, they will capture both QTLs.

## Materials and methods

### Plant material and phenotyping

The M9xR5-NZ genetic mapping family was a 133 plant subset of the population of 146 plants used to construct a genetic linkage map for apple rootstocks described by Celton et al. [48]. The inoculation of the IxR5-DE family of 132 plants in 2005 and 2006 with the German strain Ea222_JKI has been described by Peil et al. [22]. Phenotyping with New Zealand *Ea* strain ICMP11176 [87] at a bacteria concentration of 10^9^ cfu/ml was performed on 123 and 93 plants in 2006 and 2007, respectively, with 83 plants having been phenotyped in both years. The R5-DE accession was obtained from the Fruit Genbank Dresden-Pillnitz (Accession APF0409) [88]. There are no records of the importation of either the R5-DE or the R5-NZ accessions. Phenotypic evaluation of susceptibility to fire blight of both progenies was performed according to Peil et al. [22]. The number of replications varied from four to 10 plants, with many plants having two shoots, in the M9xR5-NZ family and one to 12 plants per progeny for the IxR5-DE family. A third population, O3xR5-US (R5-US = USDA-ARS PI588825 imported from Canada) of 192 seedlings was inoculated with *Ea* isolate Ea273 and E2002a, phenotypic evaluation and statistical analysis was performed according to Fazio et al. [30].

### Genetic marker analysis

A genetic comparison of the three R5 accessions was performed with several SSRs located within or immediately adjacent to the fire blight resistance QTL mapped to the upper part of LG3 [22]. These included NZmsMdMYB12, CH03e03, CH03g07 and two new markers derived bioinformatically from the apple WGS [33] located distal in the genome sequence to NZsnEH034548 and NZsnEB151679, respectively (Table1). Physical distances on the apple whole genome sequence were derived from the reference primary assembly held in the PFR genome browser, in which a 200,000 bp gap has been inserted between scaffolds. Primers designed from whole genome sequence were: NZmsMDC007176.537 forward primer: 5’ TTGCTGCCTTTAGTTTGTCCT 3’, reverse: 5’ TCACATCTTTGGGTGGTTCA 3’ and NZmsMDC018101.293 forward primer: 5’ CTCTTCCTACATTGCCCAACA 3’, reverse: 5’ GCAGCTCTTCCCACATCTTT 3’. Polymerase chain reaction (PCR) and product analysis was performed as specified by Gharghani et al. [89], but using a Hybaid MBS Satellite 0.5 G Thermal Cycler. The allele size was calculated based on internal standards using Peak Scanner (version 1.0) software (PE Applied Biosystems). To synchronize the allele detection process across individuals and loci, we identified the last stable peak on the chromatogram as an allele. All marker analyses were duplicated.

### Putative fire blight resistance gene selection

‘Fire blight unigenes’ and ‘non-fire blight unigenes’ were assembled using Phrap [90]. To identify ESTs unique to *Ea*-challenged tissue, we selected cDNA suppression subtractive hybridization (SSH) [41] and cDNA-AFLP ESTs [42] with the worst BLAST match (highest E values) to non-fire blight unigenes*.* To identify SSH and cDNA-AFLP ESTs previously observed in fire blight-challenged tissue, we selected ESTs with the best BLAST match to fire blight unigenes, SSH ESTs or cDNA-AFLP ESTs. Similarly, to identify ESTs with significant similarity to *Arabidopsis* genes regulated in response to bacterial challenge or by systemic acquired resistance, BLAST comparisons were conducted using 2800 *Arabidopsis* genes known to be regulated in response to bacterial challenge [39] and SSH ESTs downloaded from the GenBank that were isolated from *Arabidopsis* challenged with avirulent *Pseudomonas syringae*, virulent *P. syringae* or salicylic acid [91]. In addition, some putative fire blight resistance genes were chosen on the basis of bioinformatic searches in the PFR bioinformatics database [43] for sequences that have been described previously as genes for resistance to fire blight or were associated with disease resistance in general.

### Development of markers derived from putative fire blight resistance genes and genetic mapping

Markers were developed from the DNA sequences of ESTs, based on bioinformatic searches for SSRs and single nucleotide polymorphisms (SNPs) in the PFR database, using the automated bioinformatics search tool described by Newcomb et al. [43]. PCR primer pairs around the sequence of interest were developed using Primer 3 (http://frodo.wi.mit.edu/primer3/input.htm). Primer sequence, Genbank accession numbers and annotation notes are given in Additional file 1: Putative fire blight resistance genes. The new SSR and SNP markers developed were prefixed by ‘NZms’ and ‘NZsn’, respectively, followed by the Genbank accession number. The theoretical melting temperature of the primers was approximately 60°C and amplification product length was between 100 and 400 bp for SSR-based markers and 80 to 200 bp for SNP-based markers. PCR amplifications were performed as described by Gianfranceschi et al. [92], with the cycling modifications described by Celton et al. [48]. All markers were screened initially over the M9xR5-NZ bin mapping set [48]. The position of each new marker was assigned by visual inspection with respect to framework markers. Where markers were not able to be allocated to a bin in this population, owing to lack of polymorphism or difficulty in interpreting analytical traces, screening was repeated using the bin mapping set for the framework genetic map of ‘Royal Gala’ x A689-24 (RGxA689) [49]. Genotyping was performed for SSR-based markers using a CePro 9600TM (Combisep, Ames, IA, USA) capillary electrophoresis system [48]. SNP-based markers were analyzed by high resolution melting (HRM) analysis on a LightCycler® 480 (Roche Diagnostics) [49]. The map location of the markers derived from putative resistance genes that bin-mapped to the approximate location of known fire blight resistance QTLs was then verified in the M9xR5-NZ or RGxA689 populations (94 and 86 seedlings, respectively), using JoinMap version 3.0 [93] with a minimum LOD score of 4 for grouping and the Kosambi function. The merkers derived from resistance genes that mapped to the upper half of LG3 in R5-NZ were subsequently mapped in the IxR5-DE [22]. Nine putative fire blight resistance genes that were unable to be mapped genetically were located bioinformatically on the ‘Golden Delicious’ WGS [33].

### Statistical analysis of phenotype data and identification of QTL for fire blight resistance

The %SLN phenotype data were logit transformed, after adding a randomly generated and equally likely value between 0 and 1% to each zero percentage to meet the assumption of standard QTL mapping methods that the residual variation follows a normal distribution with equal variances. The genotype data were the set of markers described by Celton et al. [48] for the construction of the M9xR5-NZ genetic map with the addition of the three NZsnEH034548-, NZsnEB140229- and NZsnEB151679-derived markers mapped at LOD scores ranging from 6 to 9. For the IxR5-DE population, the markers from the M9xR5-NZ map that were scorable in this population using the technology described above were utilized, with the addition of four SSR markers, NZmsMdMYB12, NZsnCN98613, NZmsCN943818 and NZmsEB139184 to fill gaps. The QTL analyses were conducted on a subset of the markers of each population’s dataset such that the markers were distributed along the R5 linkage group and were proximal to the position of known markers derived from putative fire blight resistance genes. These subsets consisted of five and six markers spanning 21.2 and 28.0 Mb, respectively, for M9xR5-NZ and IxR5-DE populations. The NZsnEH034548-, NZsnEB140229- and NZsnEB151679-derived markers were the only markers common to both populations. Genotype coding followed for that of a pseudo-backcross with the male R5 specified as the informative parent. The R/qtl package [94] (http://www.r-project.org) was used for QTL mapping. R/qtl uses three separate methods of interval mapping: EM algorithm [50]; Haley-Knott regression [95]; and the multiple imputation methods [96]. The curves shown in Figure3 are based upon the Hayley-Knott regression method using R/qtl software. LOD thresholds were computed as percentiles of maximum LOD scores by permutation [97]. The 95% confidence limit of a discovered QTL position was estimated by the 1.5 LOD support intervals. The proportion of variation explained by the QTL was estimated as:1 – 10^–2LOD/*n*^, where *n* is the sample size. Where a putative QTL was detected by CIM, interval mapping methods were used to scan for an additional QTL in the context of an additive multiple QTL model. The resulting LOD curves were presented graphically. A two-part model implemented in the R/qtl package [98] as an alternative approach to handling the presence of many zeros in the data was fitted for comparison with results obtained by CIM.

A refined version of the O3xR5-US map [99] checked against the apple genome sequence [33] that included anchor markers to Celton et al.’s [48] map was used to estimate QTLs for the O3xR5-US phenotypic data. The analysis was conducted on all parental alleles using JoinMap ^(R)^ 4.0 for a cross pollinated population that allows the estimation of the effects of maternal alleles (a and b) and paternal alleles (c and d) possible in an heterogeneous and heterozygous cross typical of apple. The iterative process leading to QTL discovery in MapQTL® 6 involved Interval Mapping (IM) using the regression algorithm followed by Automatic Cofactor Selection to select the minimum number of markers located near QTLs discovered during IM. The Multi-QTL-Model (MQM) analysis that included the effect of selected markers in the regression model for the refinement of the QTL effect was used to determine the final map location of the QTLs.

### Bioinformatic analysis and cloning of putative fire blight resistance gene EB140229 (*i.e. Mxd*RLP1)

The unigene most similar to EB140229 in various *Malus* EST assemblies was identified by local BLASTn analysis using Bioedit (v7.05.3) [100]. Similarity of possible EST and unigene coding regions to known proteins was determined by BLASTx analysis of the NCBI nr database. High scoring sequences (>175 bits) from plants representing different branches of the Magnoliophyta and eudicotyledons were chosen for further alignment using Clustal W2 v4 [101] and manual adjustment where necessary to maximize the alignment. Identification of a potential signal peptide within the coding region was performed using PSORT [70], PSIPRED [71], and Polyphobius [72] with default parameters. Stability of single amino acid substitutions was predicted by I-Mutant 2.0 [78].

To clone the predicted full length coding region of EST GenBank:EB140229, genomic DNA was isolated from leaf tissue of R5-NZ, R5-US, G.41, the highly susceptible ‘M.9’, ‘M.26’ and ‘M.27’ rootstocks, and resistant seedling AJ103 (M.9xR5-NZ), using the Nucleon™ Phytopure™ Plant DNA isolation kit (#RPN8511), GE Healthcare. The predicted coding region was amplified from genomic DNA using primers designed from the most similar unigene (forward primer: 5’ CACCATGGGAGCTGTGCGTCTTTA 3’, reverse: 5’ AAAATACAAACACCTTCACAATCT 3’ [EB140229.R1]) and Invitrogen Platinum SuperMix high fidelity TAQ (#57053). PCR products of G.41 and M.26 were directionally cloned into a Gateway entry vector using a pENTR/D-TOPO Cloning Kit (Invitrogen K2400-20) following manufacturer’s protocols and colonies containing inserts in the correct orientation were selected by colony PCR using M13(−20) F (5’ GTAAAACGACGGCCAGT 3’) and EB140229.R1 primers. PCR products of R5-US, R5-NZ, ‘M.9’, ‘M.27’ and AJ103 were non-directionally cloned using a PCR 2.1-TOPO TA Cloning Kit (Invitrogen #K4500) and clones with inserts were selected by colony PCR using M13(−20) F and M13 pUC19 R (5’ CAGGAAACAGCTATGAC 3’) primers. The DNA sequence of inserts in colony PCR-positive clones was determined by forward and reverse Sanger sequencing. Sequences were aligned using the PHRAP and ClustalW algorithms (CodonCode Aligner v.1.5.2 and DNASTAR Lasergene MegAlign v.7.0.0).

### Expression analysis of putative fire blight resistance genes EH034548 (*i.e. Mxd*Prx8) and EH034563 ( *i.e. Mxd*Prx9)

One-year-old potted apple trees of ‘M.26’ and ‘G.41’ in a growth chamber were either mock (buffer) or *Ea* inoculated by transversely bisecting leaves as described by Norelli et al. [41]. Five leaves at the apex of vigorously growing shoots were inoculated and leaf tissue samples were collected 2, 6, and 48 hpi. Temporal synchrony of sample tissue was ensured by limiting the sample tissue to a 3–6 mm wide strip of leaf tissue cut parallel to the original inoculation cut [41]. Biological replicates consisted of pooled leaf samples from an individual tree. Mock and *Ea* samples were collected from different trees, as were the 2, 6 and 48 hpi samples.

2RNA was extracted from leaf samples using the Invitrogen Plant Concert reagent (#12322-012) according to manufacturer’s protocol for small-scale RNA extractions and DNase treated using the Ambion Turbo DNA-free kit (#AM1907) following manufacturer’s protocols. To confirm that the samples were DNA-free, DNase treated RNAs were PCR amplified with elongation factor (*EF1α)* specific primers ( *EF1α* forward 5’ATTGTGGTCATTGGTCATGT3’ *, EF1α* reverse 5’CCAATCTTGTAGACATCCTG3’). Quantitative RT-PCR was run using the Invitrogen’s SuperScriptIII Platinum SYBR Green qRT-PCR with ROX kit (#11746-100) with the following target sequence primers: EH034548 forward 5’TTCAAGTCGAGGAAGGCTTG3’, EH034548 reverse 5’TCTAATCTCCCCCTCGGTTC3’, EH034563 forward 5’GCCCTTGTTTCCAGTGAGAG3’, EH034563 reverse 5’AAGGGTTTGATGATGGTGGA3’, EH009551 ( *i.e.* cytosolic ascorbate peroxidase) forward 5’TCTGATCGCCGAGAAGAACT3’, EH009551 reverse 5’AGGGAACTGTTGCTTGATGG 3’ and *EF1α* listed above. Cycling parameters for the Roche LightCycler 480 were: cDNA synthesis at 55°C for 10 min, pre-incubation at 95°C for 5 min, amplification over 45 cycles of 95°C for 15 s, 55°C for 15 s, 72°C 15 s, cooling at 40°C for 30 s, melting curve of 95°C for 5 s, 65°C for 1 min, and then slowly increasing the temperature to 97°C with continued measurement of fluorescence.

*EF1α* was used as the target sequence normalization reference [102] and fold change in transcript abundance was calculated using the 2^-ΔΔCt^ method [103]. Equal amplification efficiencies of the target and reference were validated. Calibrator in fold change calculations was target sequence amplified from RNA of mock inoculated tissue of same cultivar-hpi as *Ea* inoculated RNA sample. Standard deviation in fold change reported in figure was calculated as described by Livak and Schmittgen [103]. The null hypothesis of no difference in fold change (base 2 log) of transcript abundance between treatments was tested using a generalized linear model (GLM procedure, SAS 9.1, SAS Institute Inc, Cary, NC, USA) that tested main effect and interactions of cultivar, hpi and target sequence (putative fire blight resistance gene). To weight the importance of biological variation over technical variation, technical replicates were nested within biological replicates in calculating the error term (total of 9 replicates [3 biological x 3 technical] per target sequence-cultivar-hpi sample, total model df = 161).

## Abbreviations

AA, Amino acid; addqtl, additional QTL mapping function of R/qtl; aka, also known as; ARS, Agricultural Research Service; bp, base pair; CI, Confidence interval; DE, Germany; Ea, Erwinia amylovora; EST, Expressed sequence tag; fb-EST, ESTs differentially expressed in response to Ea challenged; G, Geneva rootstock; IxR5, ‘Idared’ x ‘Robusta 5’ mapping population; JKI, Julius Kühn-Institut; LG, Linkage group; LRR, Leucine-rich repeat; M, Malling rootstock; M9xR5, ‘Malling 9’ x ‘Robusta 5’ mapping population; MAS, Marker-assisted selection; MDP, Predicted consensus genes from the Malus x domestica whole genome sequence; NBS-LRR, Nucleic acid binding site-leucine rich repeat; NZ, New Zealand; O3xR5, ‘Ottawa 3’ x ‘Robusta 5’ mapping population; PCR, Polymerase chain reaction; PFR, New Zealand Institute for Plant & Food Research Limited; Prx, Class 3 peroxidase gene; QTL, Quantitative trait loci; R, Resistance protein; R5, Malus x robusta ‘Robusta 5’; RLK, Receptor-like kinase; RLP, Receptor-like protein; RT-qPCR, Reverse transcriptase-quantitative PCR; US, United States of America; USDA, US Department of Agriculture; WGS, Whole genome sequence; %SLN, Percent of current season’s shoot length that became necrotic following inoculation with E. amylovora.

## Misc

Susan E Gardiner and John L Norelli contributed equally.

## Author’s contributions

SEG participated in the experimental design, and the genetic analysis of R5 accessions, as well as the selection of putative fire blight resistance genes and development of molecular markers from putative resistance genes. She participated in identification of fire blight resistance QTLs in M9xR5-NZ and IxR5-DE, and in drafting the manuscript. JLN participated in the experimental design, participated in the selection of putative resistance genes, cloned and allelotyped *MxdRLP1* in apple cultivars, determined transcript abundance of peroxidase genes, and participated in drafting the manuscript. NdS conducted statistical analyses of phenotype data, identified fire blight resistance QTLs in M9xR5-NZ and IxR5-DE, and participated in drafting the manuscript. GF developed the O3xR5-US mapping population, developed molecular markers and genetic linkage map for O3xR5-US, phenotyped O3xR5-US for fire blight resistance and identified fire blight resistance QTLs in O3xR5-US. AP developed IxR5-DE mapping population. MM participated in the experimental design, participated in the selection of putative resistance genes and located putative resistance genes within the apple genome. MH phenotyped M9xR5-NZ for fire blight resistance and participated in phenotyping of IxR5-DE. DB and CC developed molecular markers for putative resistance genes and developed genetic linkage maps for M9xR5-NZ and IxR5-DE populations. CW performed the genetic analysis of R5 accessions. YW participated in development of molecular markers and genetic linkage map of O3xR5-US. CLB participated in the selection of putative resistance genes, conducted bioinformatics analyses of *MxdRLP1*, and participated in drafting the manuscript. AMB conducted bioinformatics analyses to develop criteria used to select putative resistance genes and assembled fire blight unigenes. J-MC constructed the framework genetic linkage map of R5-NZ. KR phenotyped IxR5-DE for fire blight resistance. HSA participated in the experimental design and phenotyping of O3xR5-US for fire blight resistance. VGMB developed the M9xR5-NZ mapping population and participated in drafting the manuscript. All authors read and approved the final manuscript.

## Supplementary Material

Additional file 1 Putative fire blight resistance genes. Selection of candidate resistance genes for fire blight and their mapping by: SNP (NZsn) or SSR (NZms) markers in ‘Malling 9’ x ‘Robusta 5’ or ‘Royal Gala’ x A689-24 populations (worksheet ‘A: mapped in populations’), or bin-mapping in either of above populations (worksheet ‘B: bin mapped’), or by physical positioning within the whole genome sequence of ‘Golden Delicious’ with 200kbp gaps inserted between scaffolds [104] (worksheet ‘C: physical position”). Columns A = EST GenBank accession number; B = Description: Source of EST (project, cultivar derived from, response to *Erwinia amylovora* ( *Ea*) challenge); followed by annotation (sequence description [Genbank accession number, BLASTx similarity]); C = Selection criteria including citation for inference from scientific literature, D = Marker name; E = PCR primer sequences of marker; and F = Genetic map location and physical position in WGS. Followed by Footnotes and Literature Citations. Click here for file

Additional file 2 Fire Blight Unigenes. Unique contigs (fire blight unigenes) assembled from 5,395 apple ESTs identified from *Ea*-challenged leaves of moderately resistant ‘Red Delicious’ and highly resistant ‘Geneva 41’ apple rootstock [47]. Worksheet ‘ad file 2’ Columns A = contig number, B = bp length of contig, C = number of EST sequences in contig, D = average EST sequence length and E = contig consensus sequence. Worksheet ‘Fasta’ can be used to assemble sequences in FASTA format. Click here for file

Additional file 3 Predicted coding regions within fire blight resistance QTL on Linkage Group 7 of ‘Robusta 5’ (R5-US) accession. Homologues of predicted coding regions within fire blight (fb) resistance QTL on Linkage Group 7 of R5-US and their detection in fb transcript profiling data. Key to row fill color: gray = could play a role in host-pathogen interactions based upon annotation, light-orange = detected in at least one fb transcript profiling experiment, mid-orange = fb candidate resistance gene based upon annotation and detection, dark-orange = likely fb candidate resistance gene based upon multiple lines of evidence. Columns: A = Predicted coding sequence ID (WGS MDP#); B-E = WGS physical location in Genome Database for Rosaceae (GDR) G-Browse: B = Scaffold #, C = transcript start, D = transcript stop, E = transcript length; F = number of fb transcript profiling databases detected in; G = transcript database detected in; H-L = best functional annotation determined from GDR ‘Malus_x_domestica.v1.0_gene_pep_function_101210.formated.xls’; M-Q = Arabidopsis homologue determined from GDR ‘Malus_x_domestica.v1.0_predicted Arabidopsis homologs.xls’; R-V = Swiss Prot homologue determined from GDR ‘Malus_x_domestica.v1.0_gene_pep_uniprot_sprot_blastp_100610.formated.xls’; W-AA = TREMBL homologue determined from GDR ‘Malus_x_domestica.v1.0_gene_pep_uniprot_trembl_blastp_100610.formated.xls’. In all cases, GDR annotation columns list match ID, organism, protein description, percent identity and BLASTP E value. Column shading denotes data from different GDR annotation databases. Click here for file
